# Fluorescent PCDTBT Nanoparticles with Tunable Size for Versatile Bioimaging

**DOI:** 10.3390/ma12152497

**Published:** 2019-08-06

**Authors:** Srujan Cheruku, Lien D’Olieslaeger, Nick Smisdom, Joeri Smits, Dirk Vanderzande, Wouter Maes, Marcel Ameloot, Anitha Ethirajan

**Affiliations:** 1 Nanobiophysics and Soft Matter Interfaces group (NSI), Institute for Materials Research (IMO-IMOMEC), UHasselt—Hasselt University, 3590 Diepenbeek, Belgium; 2 Biomedical Research Institute (BIOMED), UHasselt—Hasselt University, 3590 Diepenbeek, Belgium; 3 Design & Synthesis of Organic Semiconductors (DSOS), Institute for Materials Research (IMO-IMOMEC), UHasselt—Hasselt University, 3590 Diepenbeek, Belgium; 4 IMEC, Associated lab IMOMEC, 3590 Diepenbeek, Belgium

**Keywords:** conjugated polymer nanoparticles, mini-emulsion, bio-imaging, raster image correlation spectroscopy

## Abstract

Conjugated polymer nanoparticles exhibit very interesting properties for use as bio-imaging agents. In this paper, we report the synthesis of PCDTBT (poly([9-(1’-octylnonyl)-9H-carbazole-2,7-diyl]-2,5-thiophenediyl-2,1,3-benzothiadiazole-4,7-diyl-2,5-thiophene-diyl)) nanoparticles of varying sizes using the mini-emulsion and emulsion/solvent evaporation approach. The effect of the size of the particles on the optical properties is investigated using UV-Vis absorption and fluorescence emission spectroscopy. It is shown that PCDTBT nanoparticles have a fluorescence emission maximum around 710 nm, within the biological near-infrared “optical window”. The photoluminescence quantum yield shows a characteristic trend as a function of size. The particles are not cytotoxic and are taken up successfully by human lung cancer carcinoma A549 cells. Irrespective of the size, all particles show excellent fluorescent brightness for bioimaging. The fidelity of the particles as fluorescent probes to study particle dynamics in situ is shown as a proof of concept by performing raster image correlation spectroscopy. Combined, these results show that PCDTBT is an excellent candidate to serve as a fluorescent probe for near-infrared bio-imaging.

## 1. Introduction

Over the past decade, conjugated polymer nanoparticles (NPs) have gained prominence in the bio-imaging field as an attractive class of fluorescent probes because of their interesting photoluminescence properties. They have a high absorption cross-section, high radiative rates, excellent photostability, non-blinking behavior and less susceptible to leaching as compared to small molecular weight organic dyes [1,2]. Furthermore, most of them are also biocompatible, in contrast to quantum dots [3,4]. In the last years, mainly polyfluorene [5], MEH-PPV (poly[2-methoxy-5-(2’-ethylhexyloxy)-1,4-phenylene vinylene]), CN-PPV (cyano-poly(p-phenylene vinylene)) and PPE (poly(p-phenylene ethynylene)) nanoparticles have been studied for biomedical applications [6,7,8,9,10]. Recently, different PPV NPs, i.e., MDMO-PPV (poly[2-methoxy-5-(3′,7′-dimethyloctyloxy)-1,4-phenylene vinylene]) and CPM-MDMO-PPV (2-(5’-methoxycarbonylpentyloxy)-5-methoxy-1,4-phenylene vinylene), were tested in our group [11]. As the latter introduce carboxyl groups at the NP surface, the coupling of biomolecules to the nanoparticles was successfully demonstrated [12]. 

The main drawback when applying these materials for bio-imaging is that they require excitation and emit in the visible region (390–700 nm), resulting in a lower sensitivity and specificity for bio-imaging and tracking applications. In general, for fluorescence bio-imaging, scattering and absorption of photons by biological tissue is of major concern, as both incoming (excitation) and outgoing (emission) light are affected. In this regard, fluorescent probes active in the near-infrared (NIR) region (~700–900 nm) have triggered a lot of interest, as the NIR range coincides with the optical window of biological tissues, offering a larger penetration depth into biological tissues and a good spectral separation from cellular autofluorescence [5,13,14,15,16,17,18,19]. Recently, some research groups investigated conjugated polymers emitting in the NIR window for bio-imaging. Geng et al. synthesized nanoparticles out of the donor-acceptor copolymer PFTTQ (poly[9,9-bis(4-(2-ethylhexyl)phenyl)fluorene-alt-co-6,7-bis(4-hexyloxyphenyl)-4,9-di(thiophen-2-yl)thiadiazoloquinoxaline]), absorbing and emitting in the NIR region, using a precipitation approach [13]. Wu et al. synthesized squaraine-based polymer dots with a large Stokes shift and narrow band emissions in the NIR range [14]. Furthermore, Hong et al. synthesized a water-soluble donor-acceptor polymer pDA (poly(benzo[1,2-b:3,4-b′]difuran-alt-fluorothieno[3,4-b]thiophene)) and demonstrated in vitro and in vivo biological imaging in the NIR biological window using this polymer [15]. Moreover, also Förster resonance energy transfer strategies were applied to design conjugated materials emitting in the NIR [16,20].

So far, donor-acceptor or push-pull type conjugated polymers are mainly studied for their use in organic electronics. In the field of organic photovoltaics (OPV), they are of particular interest because of their small bandgap, as this increases their absorption overlap with the solar spectrum [21,22]. As the absorption spectrum shifts to the red region, the same holds for the emission, and this bathochromic shift can extend into the NIR, which renders these materials suitable for imaging in the NIR biological window. Nevertheless, only very limited reports on the use of such polymers for bio-imaging exist to date. 

Among the different donor-acceptor copolymers, PCDTBT is an established workhorse material in organic photovoltaics and its optical properties have been studied extensively for this application [23,24]. However, despite its interesting photoluminescence features, to our knowledge there have been no previous reports of PCDTBT being studied for use in bio-imaging applications. This can probably be attributed to the fact that this polymer is very hydrophobic and therefore formulations are required that allow to use this material in an aqueous biological environment. Usually, techniques such as mini-emulsion or reprecipitation are employed to formulate water-based conjugated nanoparticle dispersions. However, a lot of the push-pull copolymers have solubility issues in respect to not being soluble in most low-boiling organic solvents. This complicates the synthesis of nanoparticles by mini-emulsion [1,25,26,27] reprecipitation [1,28,29,30], as both techniques require a good solubility in low-boiling solvents, with an additional requirement that the solvent is also miscible with water in case of the reprecipitation method. Recently, our group has already reported the synthesis of PCDTBT particles systematically doped with a different ratio of PC71BM ([6,6]-phenyl-C71-butyric acid methyl ester) by adapting the mini-emulsion technique of Landfester et al [25,27]. and using a high-boiling solvent for the nanoparticle formulation [31]. In the latter work, the particles were successfully used for photovoltaic applications. 

In the current work, we further adapted our previously reported synthesis method [31] to make PCDTBT nanoparticles of different sizes. Interchain interactions are known to play a crucial role in determining the optical characteristics of conjugated polymer aggregates as compared to their molecularly dissolved counterparts [32,33,34]. In addition to its influence on the optical properties, the size of the NPs is also a very important considering biological applications. The size of the NPs has been shown to influence cellular uptake. Endocytosis is among the main biological processes responsible for the active internalization of NPs. Endocytosis occurs through processes like phagocytosis, receptor mediated endocytosis, adsorption or through one of a variety of alternate uptake pathways. It has been previously determined that the optimal size of NPs for active endocytic uptake ranges from about 25–50 nm [35]. NP size is also known to be one of the important determinants of their cytotoxicity, this is due to the higher surface area ratios (compared to their mass) of smaller NPs increasing their chances of their interaction with the biological milieu. Besides uptake and cytotoxicity, the fate (biodistribution and clearance) of NPs is also determined by their size among other factors [35,36,37]. We have previously reported the size dependent properties of PPV NPs which have emission in the visible spectrum (λmax 590–640 nm) [11]. 

In addition to varying the size of the PCDTBT NPs, their optical properties were carefully characterized with an eye towards their use in bio-imaging. Further the biocompatibility and cellular uptake of the NPs was investigated using the human lung cancer carcinoma A549 cell line. Raster image correlation spectroscopy (RICS) is a noninvasive image analysis technique that can be used to measure the diffusion coefficient and concentration of fluorescently labeled entities using a laser scanning microscope. The ability to monitor the diffusion characteristics of the PCDTBT NPs in situ using RICS was also tested in water as an initial proof on concept, to demonstrate the potential applicability of these particles for bio-imaging and tracking dynamics in biological environments. To the best of our knowledge, such studies for PCDTBT NPs—which have fluorescence in the NIR biological window—do not exist to date, and this report provides a deeper insight about the suitability of these particles as bio-imaging probes.

## 2. Materials and Methods

### 2.1. Materials

PCDTBT (Mw = 79 kDa, D = 2.4) was purchased from Solaris Chem Inc. (Vaudreuil-Dorion, QC, Canada). Merck Millipore (Cork, Ireland) delivered Amicon Ultra-4 centrifugal filters (MWCO = 3.0 kDa). Cryptocyanine was used as a reference for the photoluminescence quantum yield measurements. Sodium dodecyl sulfate (SDS) and 1,2-Dichlorobenzene (o-DCB, 99.0%) were purchased from Sigma Aldrich (Steinheim, Germany). Alamar blue and the modified eagle’s medium with GlutaMAX and penicillin/streptomycin were purchased from Life technologies (Ghent, Belgium). Non-heat inactivated fetal bovine serum was obtained from Biochrom AG (Berlin, Germany).

### 2.2. Nanoparticle Synthesis

**Figure 1:**
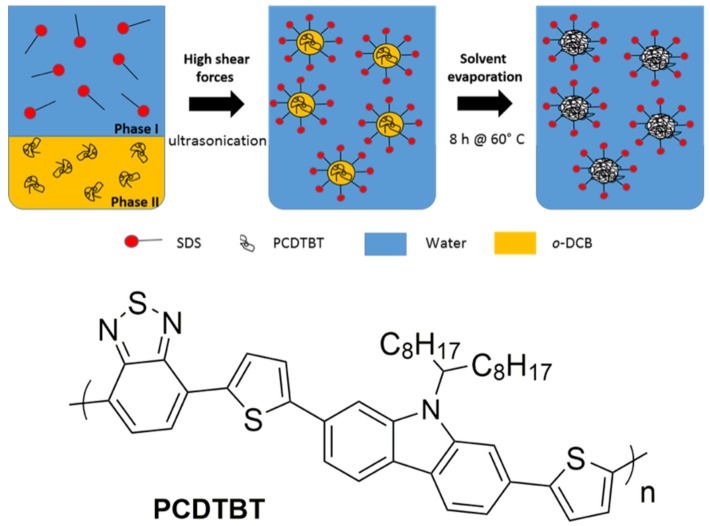
(**top**) Schematic illustration of the PCDTBT NP synthesis. (**bottom**) Chemical structure of PCDTBT.

PCDTBT nanoparticles were synthesized by slightly altering the previously reported synthesis procedure using the combined mini-emulsion and solvent evaporation technique in combination with a high-boiling solvent [31]. Different sizes of nanoparticles were obtained by varying the concentration of the dispersed phase. Varying amounts of PCDTBT were dissolved in 2.0 g o-DCB at 80 °C in a nitrogen environment (Table 1). Subsequently, an SDS solution of 11 mg SDS in 2.64 g water (0.4 wt%) was added to the organic phase. After stirring for 1 h, the mini-emulsion was obtained by ultrasonication for 180 s (30 s pulse, 20 s pause) at 60% amplitude using a Branson Mylar 1/8” tip under ice cooling. Immediately after the sonication process, the mini-emulsion was transferred to a round bottom flask with a wide neck and left for 8 h at 60 °C for solvent evaporation. Additional water was added every hour to the emulsion to compensate for the loss during evaporation. The NP samples were washed with Milli-Q ultrapure water as needed to remove the excess surfactant using centrifugal filter devices. The synthesis procedure is summarized in Figure 1.

### 2.3. Transmission Electron Microscopy (TEM)

The dispersions of nanoparticle were diluted and drop cast on a carbon coated copper grid. TEM imaging was performed using a TECNAI spirit TEM from FEI (Zaventem, Belgium) operating at 120 kV.

### 2.4. Dynamic Light Scattering (DLS)

The size, size distribution and zeta potential of all NPs was characterized by DLS in water using a Brookhaven Instruments (Waddinxveen, The Netherlands) Zetapals.

### 2.5. Stationary UV-Vis Absorption and Fluorescence Spectroscopy

The UV-Vis absorption spectra of the NPs were measured using a Cary5000 Scan UV-Vis-NIR spectrophotometer from Agilent Technologies (Diegem, Belgium). The emission spectra were obtained using an Yvon FluoroLog-3 spectrofluorometer supplied by Horiba (Lier, Belgium), with correction for the wavelength dependence of the throughput and sensitivity of the detection channel. 

The photoluminescence quantum yields (PLQYs) of the polymers in o-DCB and the nanoparticles in water were measured using the fluorescence quantum yield (FQY) cryptocyanine in ethanol as a standard [38]. Five dilutions were prepared for all samples as well as for the standard. The most concentrated one had an absorbance of 0.1 at an excitation wavelength of 540 nm. The absorption coefficients of the materials were calculated using Lambert-Beer’s law by changing the concentration of the nanoparticles in water or the polymers in o-DCB. Emission spectra were obtained for all samples, after which the absorption versus the integral of the emission spectra for each of dilutions and samples were plotted and fit. The slope values (m) of the fit as well as the refractive indexes (η) of the solvents were used to determine the PLQYs of the samples using formula [11]:

$$PLQY_{\mathrm{sample}}=\;FQY_{\mathrm{standard}}\frac{m_{\mathrm{sample}}}{m_{\mathrm{standard}}}\frac{\eta_{\mathrm{sample}}^{2}}{\eta_{\mathrm{standard}}^{2}}$$

### 2.6. Cell Cultures

Adenocarcinomic human alveolar basal epithelial cells (A549) routinely used in the group were used for the cell studies [11,34,39,40]. A549 cells (European Collection of Authenticated Cell Cultures, Wiltshire, UK) were cultured in modified Eagle’s medium (MEM) with GlutaMAX, supplemented with 10% non-heat inactivated fetal bovine serum (FBS) with 1% penicillin/streptomycin added. They were incubated at 37 °C with 5% CO_2_ and subcultured on reaching about 80% confluence.

### 2.7. Cytotoxicity Studies

The cytotoxicity of the various PCDTBT NPs was assessed using the Alamar blue assay. A549 cells were seeded at a density of 10,000 cells/well in a black 96-well plate and allowed to incubate for 24 h at 37 °C with 5% CO_2_. The NPs were then up to concentrations of 100 µg/mL and incubated for a further 24 h. Subsequently, the cells were washed with phosphate buffered saline (PBS). This was followed by the addition of the Alamar blue reagent to the wells as per manufacturer’s instructions. After an incubation for 4 h, the plate was read using a fluorescence plate reader (Tecan).

### 2.8. In Vitro Imaging

A549 cells were grown in 8 well μ-slides. The cells were subsequently incubated (individually) at 37 °C with 5% CO_2_, with NP1-5 at a concentration of 50 µg/mL for 18 h. The cells were then rinsed with PBS to remove the free NPs. Two-photon excitation fluorescence microscopy (TPEM) was conducted at room temperature using an LSM 510 META (Zeiss, Zaventem, Belgium) confocal laser scanning microscope (CLSM) on an inverted Axiovert 200 M motorized frame (Zeiss). The microscope was fitted with a LD C-Apochromat 40×/1.1 W Corr UV-VIS-IR water immersion objective. The NPs were excited using a femtosecond pulsed titanium-sapphire laser (MaiTai DeepSee, Spectra-Physics, Santa Clara, CA, USA) tuned to 950 nm. The emission was then routed through a 650 nm low pass filter before being captured using a non-descanned detector (NDD). The images on the transmission channel were also captured on a separate detector mounted to the microscope turret. The internalization of the NPs by the cells was confirmed by acquiring z-stacks over the volume of cells (optical slice thickness = 1 µm).

### 2.9. Raster Image Correlation Spectroscopy

For raster image correlation spectroscopy (RICS), a series of 100 images of a dispersion of the PCDTBT NPs in water were acquired with a pixel dwell time of 16.38 µs using an LSM 880 (Zeiss) confocal laser scanning microscope on an inverted Axio observer motorized frame. A 256 × 256 pixel sized image, with a pixel size of 83 nm was acquired. The microscope was fitted with a Plan-Apochromat 20×/0.8 objective. A femtosecond pulsed titanium-sapphire laser (MaiTai DeepSee, Spectra-Physics) tuned to 810 nm was used as the excitation source for TPEM. The fluorescence emission was then channeled through a 690 nm low pass filter. The BiG-2 (Zeiss) non-descanned detector was used to capture the images. Arbitrary-region RICS (ARICS) analysis was applied to determine the diffusion coefficient [41]. Aggregates (if any) were masked out using an absolute intensity threshold, while the remaining areas of the image were left unprocessed. The analysis and fit were performed using the PAM software package using the Microtime Image Analysis and MIAFit modules [42]. 

## 3. Results and Discussion

### 3.1. Nanoparticle Synthesis and Characterization

Different sizes of PCDTBT nanoparticles were synthesized using the combined mini-emulsion and emulsion/solvent evaporation technique employing high-boiling solvents [31]. For a given dispersed phase/surfactant ratio used, different sizes of PCDTBT NPs were made by altering the concentration of the polymer in the dispersed phase. The characteristics of the different particles are summarized in Table 1. 

**Figure 2:**
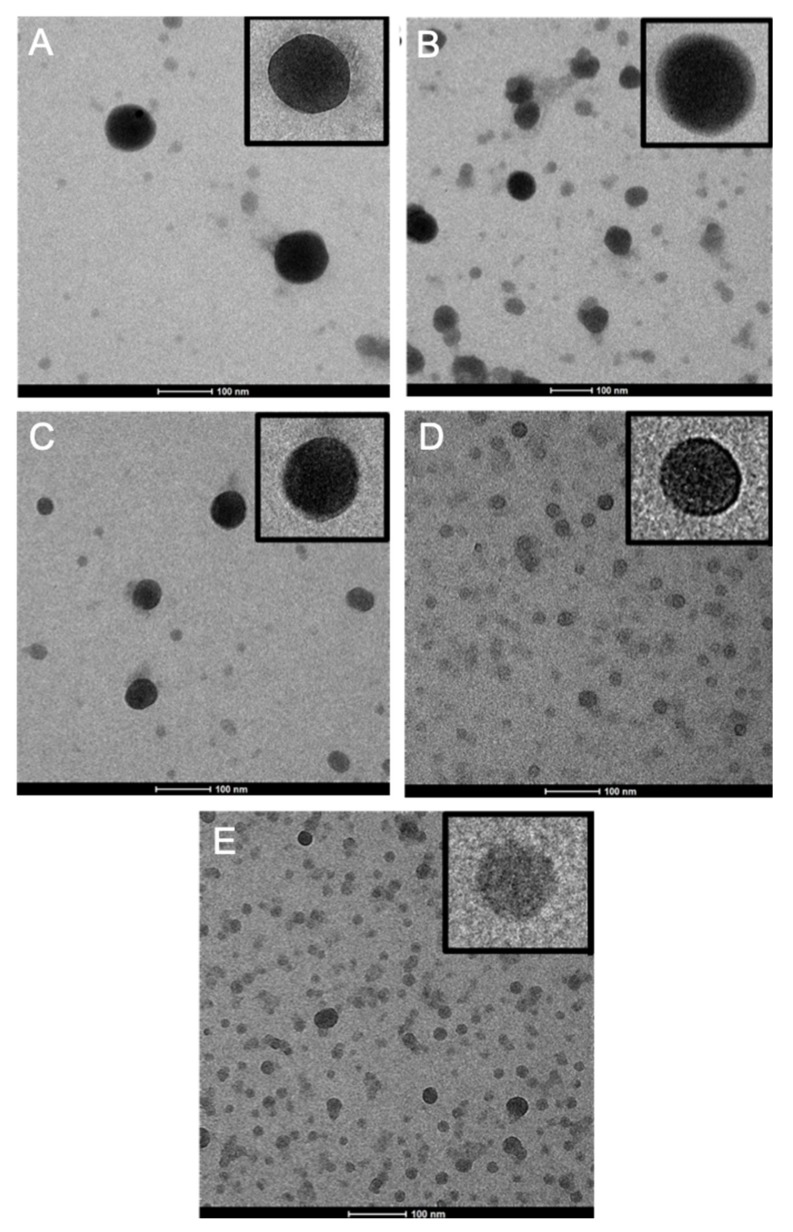
TEM micrographs of the different sized nanoparticles: (**A**) NP1, (**B**) NP2, (**C**) NP3, (**D**) NP4, and (**E**) NP5. The inset shows the morphology of a single particle in an enlarged view.

It can be seen that lowering the concentration of the polymer in the dispersed phase results in smaller particles. The polydispersity index (PDI) increases upon decreasing particle size. Especially the two smallest particles (NP4 & NP5) show a broader size distribution. As the amount of surfactant used during the synthesis was the same for all particles, there might be aggregate formation in case of the smaller particles, since there is an increase in the interfacial area of the NPs that needs to be covered by the surfactant molecules. In general, all NP dispersions exhibited a good stability and the zeta potential values for all samples, after excess surfactant removal by washing, ranged between −21 and −38 mV. The solid content of all NP dispersions, measured immediately after the synthesis, is quite low because additional water was added during the solvent evaporation step of the synthesis. In Figure 2, the TEM micrographs of the different particles are shown. Here, the particles appear smaller as compared to the values obtained by DLS, which is to be expected as the latter measures the hydrodynamic radius.

### 3.2. Size-Dependent Optical Properties

**Figure 3:**
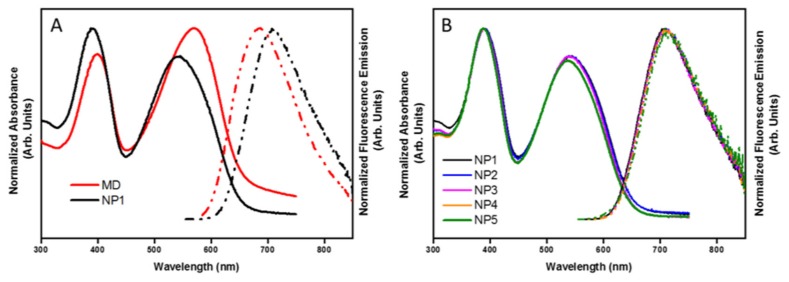
UV-Vis absorption (solid lines) and fluorescence (dashed lines) spectra of (**A**) PCDTBT in NP (NP1) and MD form, and (**B**) the differently sized PCDTBT NPs (NP1, NP2, NP3, NP4, NP5). The UV-Vis absorption spectrum of the NPs is hypsochromically shifted and fluorescence spectrum bathochromically shifted, compared to its MD form.

The optical properties of the PCDTBT NPs are crucial to define their bio-imaging applicability. Therefore, the optical properties of the NPs were studied as a function of their size using different spectroscopic techniques. The absorption and emission spectra of all PCDTBT NPs and molecularly dissolved PCDTBT (in o-dichlorobenzene) were obtained using UV-Vis absorption and fluorescence spectroscopy (Figure 3). The polymer nanoparticles have two characteristic absorption peaks with maxima around 390 and 540 nm, and an emission peak at 708 nm. The emission wavelength of 708 nm falls within the NIR biological window (680–900 nm), offering a good spectral separation from cellular autofluorescence. The absorption and emission spectra of the NPs are hypsochromically (blue) and bathochromically (red) shifted, respectively, as compared to those of the polymer chains molecularly dissolved in o-dichlorobenzene (Figure 3A). These shifts are consistent with the likely formation of H-type aggregates (sandwich packing of the polymer chains) when the polymer is formed into NPs [43]. A large Stokes shift and a low PLQY are characteristic for these aggregates (Table 2). In Figure 3b, the absorption and emission spectra of the differently sized NPs are shown. Although the different samples do not show large differences, very small changes can be observed between the different NPs, with the smaller particles exhibiting a slightly larger hypsochromic shift in the absorption and a bathochromic shift in the emission spectra. These small shifts can be attributed to the fact that the polymer chains can be packed in a more ordered way in smaller particles during the synthesis. When the dispersed phase is more diluted, the polymer chains are freer to move inside the droplets and the polymer chains have more time to place themselves in the favorable sandwich packing.

Table 2 summarizes the optical characteristics of PCDTBT in the NPs of different size and in the MD form. A strong decrease in PLQY was observed, from 41% for the MD polymer to around 3–6% for the different NPs. This significant lowering of the PLQY can be attributed to the aggregation of the polymer chains in the NPs, resulting in a non-radiative interchain pathway for the excitation energy decay. Other reports show that the PLQY decreases in larger particles due to the higher probability of interchain interactions [44,45]. Contrary to the reports in literature, our PLQY values are not decreasing with larger particle sizes. As stated previously, PCDTBT likely forms H-type aggregates when formulated into NPs. In these aggregates, the excited state radiative decay is suppressed, resulting in a very low PLQY yield. A trend can be seen for the differently sized NPs. The polymer chains are more ordered and more likely to form H aggregates in case of the smaller particles, resulting in a lower PLQY. Although the PLQY values of the PCDTBT NPs are quite low, their molar extinction coefficients, ~1 × 10^6^ −2 × 10^6^ M^−1^ cm^−1^ for all size ranges (with small variations), are quite large. As such, the fluorescent brightness, which is the product of the PLQY and the molar extinction coefficient, though not as high as those of small molecule organic dyes is relatively high compared to other conjugated polymer NPs [11]. This, when combined with other superior optical properties of conjugated polymer NPs [1,2] render these PCDTBT NPs suitable candidates as fluorescent probes for imaging within the NIR biological window.

### 3.3. Biocompatibility Studies

**Figure 4:**
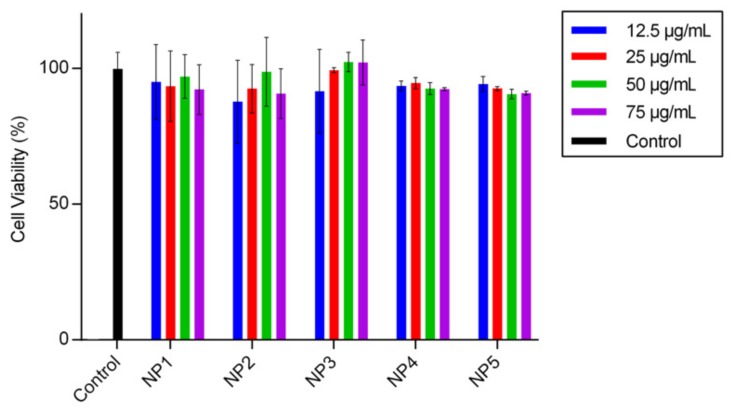
Dose-dependent cytotoxicity of the different PCDTBT NPs after 24 h of exposure, as determined by the Alamar blue assay on A549 cells, showing no significant cytotoxicity. Error bars show the standard deviations of the measurements (n = 3).

To use the PCDTBT NPs in bio-imaging applications, the particles should obviously not be cytotoxic. Therefore, their biocompatibility was tested using an Alamar blue assay on A549 human lung carcinoma cells. The results of the cell viability assay are shown in Figure 4. In general, all PCDTBT NPs show low cytotoxic effects with approximately 90% cell viability for all sizes and concentrations. This is a promising result, crucial for further advanced in vitro studies to analyze cellular dynamics and uptake mechanisms. 

### 3.4. In Vitro Imaging

**Figure 5:**
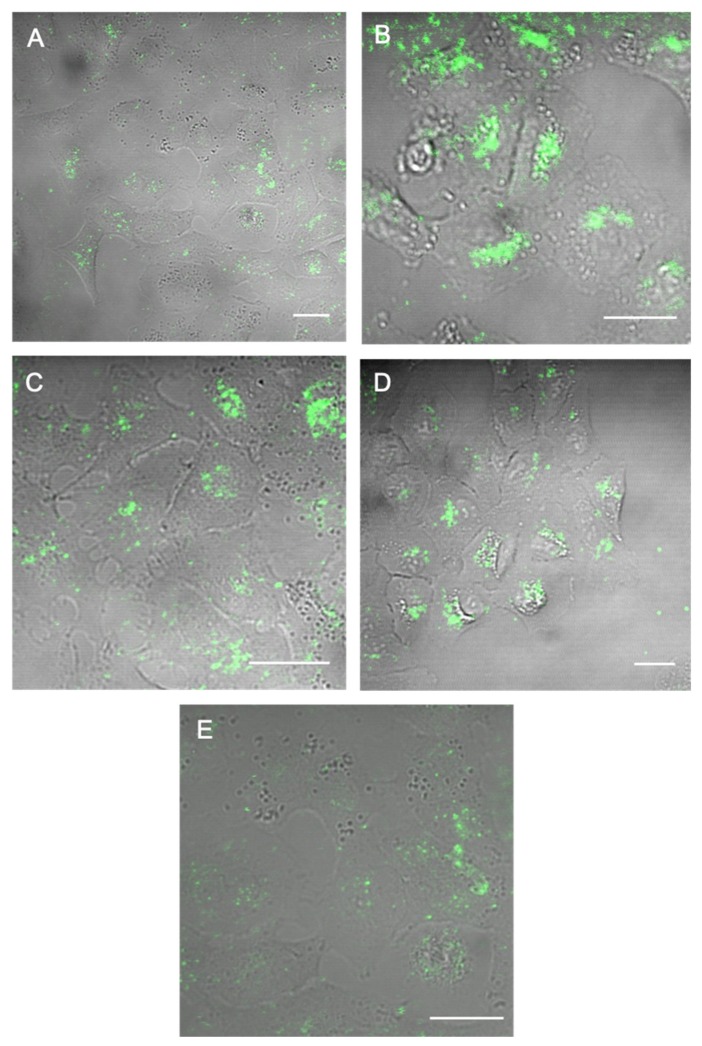
Two-photon microscopic images of A549 cells incubated for 18 h with 50 µg/mL of (**A**–**E**) NP1–NP5 respectively. The NPs are seen in green; they are superimposed on transmission images of the cells. All the NPs are internalized by the A549 cells. Scale bar: 25 µm.

To evaluate the performance of the PCDTBT NPs as fluorescent bio-imaging probes, the selected NP samples were visualized in vitro. A549 cells were chosen as a representative cell model. All the NP samples were internalized by the cells and were successfully imaged using TPEM after 18 h incubation, as shown in Figure 5. A z-stack (optical section interval: 1 µm) was also obtained (see videos included in Supplementary Materials) to distinguish NPs internalized by the cells from those bound to the outside of the cell membranes. 

### 3.5. Raster Image Correlation Spectroscopy

RICS using a laser scanning microscope enables to study the dynamics of fluorescent entities within a biological environment, such as intracellular dynamics and diffusion characteristics of NPs [46,47]. Hemmerich et al. have previously used RICS analysis to determine the diffusion characteristics of polystyrene and silica NPS in cells [48]. Intra cellular transport processes of NPs are of utmost importance to understand their cellular fate. In addition, the movement of the NPs within a cell might also provide an insight into the mechanical properties of the surrounding microenvironment [49]. However this has not been previously reported with PCDTBT NPs, as a first step to check the applicability of this technique to the particles under study, ARICS was applied to estimate the diffusion coefficients of the NPs in water (Table 3). More specifically, ARICS analysis does not require a square region of interest to be analyzed as in the case of conventional RICS analysis. This was exploited to better deal with any nonspecific aggregates that might diffuse into the measurement focal volume, by masking such aggregates by applying absolute intensity thresholds on the images [41].

**Figure 6:**
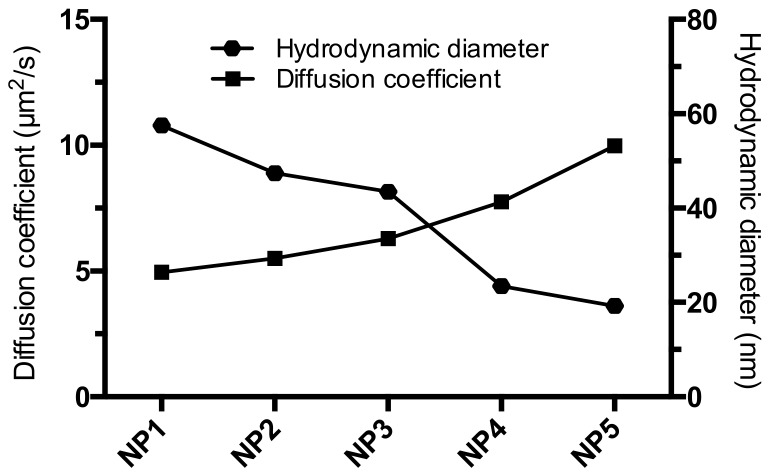
Diffusion coefficients of the different PCDTBT NP samples as measured by RICS. The diffusion coefficients increase as the hydrodynamic size (as measured by DLS) of the particles decreases.

The diffusion coefficients measured using ARICS show a decreasing trend with increasing particle size (Figure 6), as expected based on the Stokes-Einstein equation. These results serve as a proof of concept to illustrate the potential use of these particles in the RICS approach to study their dynamics in situ using their fluorescent signals.

## 4. Conclusions

PCDTBT nanoparticles of different size, ranging from 58 nm for the largest particles to 19 nm for the smallest particles (as measured by DLS), were synthesized by combining mini-emulsion-solvent evaporation method. Systematically lowering the polymer concentration in the dispersed phase resulted in smaller particles. The optical properties of the NPs were studied, which revealed that the PCDTBT NPs can be excited with two-photon excitation in the near-infrared region, hence within the NIR biological window. Moreover, it was shown that the polymer has different characteristics in the form of nanoparticles as compared to the polymer in its molecularly dissolved form. A clear hypsochromic shift in the absorbance and a bathochromic shift in the emission spectrum was observed when the polymer was formulated into nanoparticles. This corresponds to the formation of H-aggregates, resulting in a large Stokes shift (around 170 nm) and low PLQY values (in the range of 3–6%). The quantum yields showed a decreasing trend as the size of the NPs decreased. This can be attributed to the difference in arrangement of the polymer chains and thus the difference in the ratio of H aggregates in the case of the smaller particles as compared to the larger particles. In the former case, it is likely that the polymer chains are more ordered, as the chains can more freely diffuse inside the droplet, owing to the lower amount of polymer in the dispersed phase, and therefore, have a higher probability of placing themselves in the sandwich packing, forming H aggregates. Biocompatibility studies showed that for the NPs tested the cell viability remains above 90%. Furthermore, the uptake of the different particles was visualized using confocal microscopy. The use of these particles to study their dynamics in situ, with the view of extending this to probe nanoparticle-cell interactions, was also validated as a proof of concept by using RICS to study the diffusion of the particles in water. The fidelity of the reported technique to synthesize NPs of different sizes with ease, along with their high fluorescence brightness, the ability to emit in the NIR biological window and benign biological characteristics make these PCDTBT NPs excellent candidates for use as bio-imaging probes.

## Figures and Tables

**Table 1:** Physical characterization (by DLS) data of the various PCDTBT nanoparticles obtained by using different amounts of PCDTBT in the dispersed phase.

Sample	PCDTBT (mg)	Size in DLS (nm) *	Geometric Standard Deviation	PDI
NP1	25	57	1.4	0.138
NP2	15	47	1.5	0.179
NP3	10	44	1.5	0.189
NP4	5	24	1.6	0.211
NP5	2	19	1.6	0.212

* Geometric mean of the log-normal number distribution.

**Table 2:** Summary of the optical properties of molecularly dissolved (MD) PCDTBT and PCDTBT NPs of different size.

Sample	λ_max_ Excitation (nm)	λ_max_ Emission (nm)	Stokes Shift (nm)	PLQY (%)
MD	398–571	687	116	41
NP1	391–543	711	168	6
NP2	391–541	709	168	4
NP3	389–541	709	168	3
NP4	388–563	710	174	3
NP5	388–538	711	173	3

**Table 3:** Diffusion coefficients of the PCDBT NPs in water as determined by RICS analysis.

Sample	Diffusion Coefficient (µm^2^/s)
NP1	4.95
NP2	5.51
NP3	6.30
NP4	7.76
NP5	9.98

## Supplementary Materials

The following are available online at https://www.mdpi.com/1996-1944/12/15/2497/s1, Normalized absorbance and photoluminescence spectra of molecularly dissolved PCDTBT and PCDTBT NPs, videos with z-stacks of the two-photon images and a table containing the χ^2^ values of the fit for the RICS data.
