# Supplementary material for: Fluorescent PCDTBT Nanoparticles with Tunable Size for Versatile Bioimaging
Source: Materials (Basel). 2019 Aug 6;12(15):2497. doi: 10.3390/ma12152497 (PMC6695891; doi:10.3390/ma12152497)
Supplement: Supplementary file 1 [file materials-12-02497-s001.zip › materials-565017-proof done -SI/materials-565017-layout - SI.pdf]

materials

MDPI

Article

Supplementary Materials: Fluorescent PCDTBT Nanoparticles with Tunable Size for Versatile Bioimaging

Srujan Cheruku [1], Lien D'Olieslaeger [1], Nick Smisdom [2], Joeri Smits [1], Dirk Vanderzande [3,4], Wouter Maes [3,4], Marcel Ameloot [3] and Anitha Ethirajan [1,4,*]

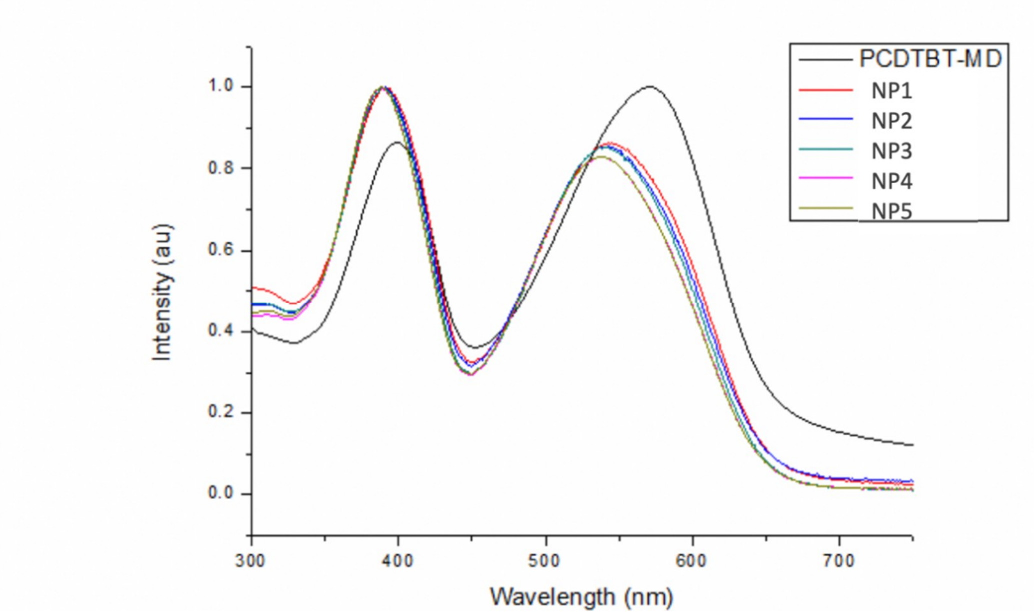

Figure S1. Normalized absorbance and photoluminescence spectra of molecularly dissolved PCDTBT and nanoparticles.

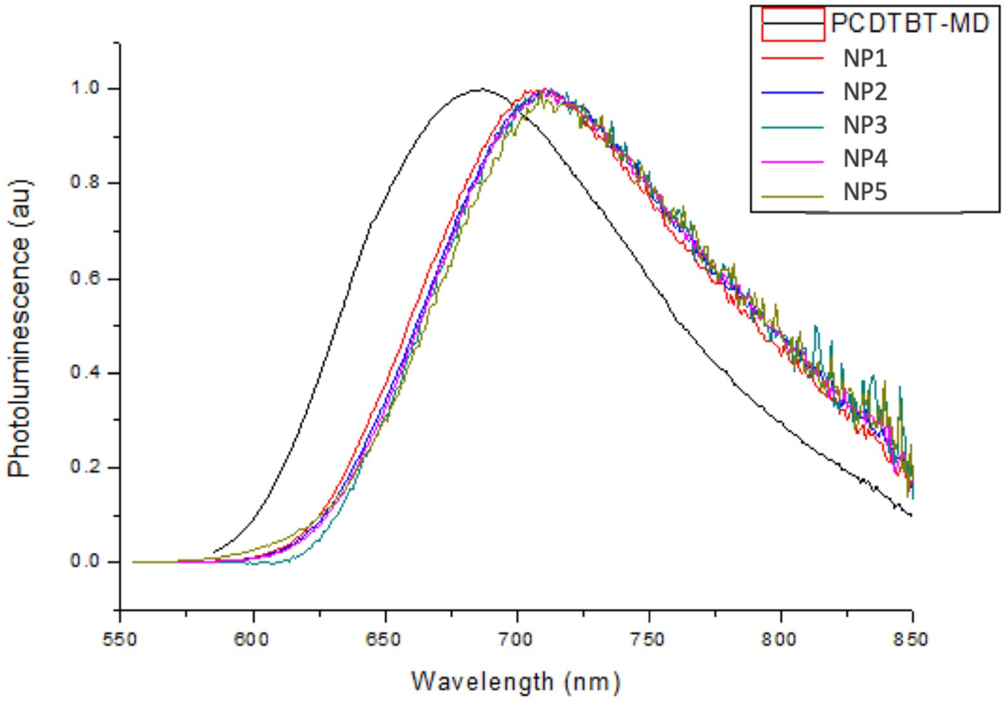

Figure S2. Normalized photoluminescence spectra of molecularly dissolved PCDTBT and nanoparticles.

Video S1. Z-stack (optical slice thickness = 1 μm) of **NP1** NPs incubated with A549 cells for 18 h. The NPs (green) are seen distributed within the volume of the cell.

Video S2. Z-stack (optical slice thickness = 1 μm) of **NP5** NPs incubated with A549 cells for 18 h. The NPs (green) are seen distributed within the volume of the cell.

Table S1. Diffusion coefficients of the PCDBT NPs in water as determined by RICS analysis with the χ^2 values of their respective fits.

**Sample**	**Diffusion Coefficient (μm²/s)**	χ^2
NP1	4.95	1.41
NP2	5.51	2.95
NP3	6.30	1.59
NP4	7.76	1.29
NP5	9.98	1.66
